# Synergistic Regulation of Transcription and Translation in Escherichia coli Revealed by Codirectional Increases in mRNA Concentration and Translation Efficiency

**DOI:** 10.1128/spectrum.02041-21

**Published:** 2022-02-09

**Authors:** Huong Le Nguyen, Marie-Pierre Duviau, Sandrine Laguerre, Sébastien Nouaille, Muriel Cocaign-Bousquet, Laurence Girbal

**Affiliations:** a TBI, Université de Toulouse, CNRS, INRAE, INSA, Toulouse, France; Northwestern University

**Keywords:** *Escherichia coli*, gene expression regulation, mRNA concentration, polysome profiling, ribosome density, ribosome occupancy, transcription, translation regulation

## Abstract

Translational regulation was investigated at the genome-scale in Escherichia coli cells. Using the polysome profiling method, the ribosome occupancy (RO) and ribosome density (RD) of different mRNA copies were determined for several hundred mRNAs during the exponential- and stationary-phases, providing the most complete characterization of such regulation in E. coli. Although for most genes, nearly all mRNAs (>90%) were undergoing translation, they were loaded with far fewer than the theoretical maximum number of ribosomes, suggesting translation limitation at the initiation step. Multiple linear regression was used to identify key intrinsic factors involved in the genome-wide regulation of RO and RD (i.e., open reading frame GC%, protein function, and localization). Unexpectedly, mRNA concentration, a factor that depends on cell physiology, was predicted to positively regulate RO and RD during the exponential- and stationary-phases. Using a set of selected genes controlled by an inducible promoter, we confirmed that increasing the mRNA concentration upon transcription induction led to increases in both RO and ribosome load. The fact that this relationship between mRNA concentration and translation parameters was also effective when E. coli cells naturally adapted to carbon source changes demonstrates its physiological relevance. This work demonstrated that translation regulation is positively controlled by transcript availability. This new mechanism contributed to the codirectional regulation of transcription and translation with synergistic effects on gene expression and provided a systemic understanding of E. coli cell function.

**IMPORTANCE** The process of gene expression is divided into translation and transcription. Considerable efforts have been made in bacteria to characterize the mechanisms underlying translational regulation and identify the regulatory factors for particular mRNAs. However, to understand bacterial physiology and adaptation, it is important to elucidate genome-wide translational regulation and examine its coordination with transcriptional regulation. Here, we provided a genome-wide picture of translational regulation in Escherichia coli. For most genes, nearly all mRNA copies were found to undergo translation but were loaded with a low number of ribosomes. We showed that mRNA concentration had a positive effect on translation regulation, linking translational regulation to transcriptional regulation as well as to cell physiology and growth conditions. The codirectional regulation of transcription and translation had synergistic effects on gene expression, contributing to E. coli cell function optimization. This finding could be used in biotechnology to optimize strategies for recombinant protein synthesis.

## INTRODUCTION

Protein synthesis is an important cellular process because the translation of mRNA into proteins is the most energy-consuming process in bacterial cells (1). A fundamental question is how and to what extent the cell controls the synthesis of proteins to optimize its function. Protein synthesis depends on the mRNA concentration (2) and is, therefore, regulated at the transcriptional level. However, mRNA and protein concentrations are only moderately correlated (between 0.36 and 0.7) (2–4), indicating additional regulation at the translational level and raising questions. Is translational regulation generally coordinated with transcriptional regulation in bacteria? Are they codirectional, with synergistic effects on gene expression or antagonistic, leading to attenuated gene expression? Deciphering the general rules of translational regulation should shed light on the coordination of transcriptional and translational regulation and provide a better understanding of bacterial physiology and adaptation processes.

Translational regulation in bacteria has not been often studied but has been identified in response to environmental cues and stress (5–8), and exponentially growing cells (9). Regulation occurs in both the initiation and elongation phases of translation by sequence-related modulation of ribosome-binding efficiency and the elongation rate. For example, ribosome binding is regulated by the level of secondary structure in the 5′ untranslated region (UTR), the strength of the ribosome binding site (RBS) (10), and the ribosome elongation rate by codon usage bias in the coding sequence (11–13). While these ribosome-related forms of regulation apply to all mRNAs, other factors are more specific and regulate the translation of subsets of mRNAs. mRNA binding of regulatory proteins and small RNAs (sRNAs) represses and activates translation initiation, for instance, by overlapping with the Shine-Dalgarno sequence and preventing the formation of inhibitory secondary structure (as exemplified for the protein CsrA and RyhB sRNA (14, 15)). Although each of the above-mentioned regulatory factors have been well studied individually, their relative importance in translational regulation at the genome-wide level in E. coli has not been quantified.

The development of high-throughput methods, such as ribosome and polysome profiling has made it possible to study translational regulation at the genome-wide level in bacteria. Genome-wide translational information is usually obtained in bacteria using the ribosome profiling method based on ribosome footprints (6, 9, 16–20). This method, which is experimentally quite simple, provides the average ribosome density (RD) per mRNA, which is defined as the total number of bound ribosomes divided by the total number of mRNA copies. However, the number of bound ribosomes differs between mRNA copies (7, 21). The specific ribosome load of each mRNA copy can only be determined using the polysome profiling method based on the physical separation of mRNA molecules in terms of the number of bound ribosomes. Moreover, polysome profiling is the only approach that can quantify ribosome occupancy (RO): the proportion of mRNA copies undergoing translation for each gene. Large-scale polysome profiling estimates of RD and RO are lacking in E. coli but are required for a genome-wide understanding of translational regulation in this model bacterium.

To determine the general rules of translational regulation and gain a better understanding of its coordination with the transcription process, we used polysome profiling to perform the first genome-wide analysis of the translational status of E. coli mRNAs in fast-and nongrowing cells. Multiple linear regression was used to identify and rank the effects of multiple parameters on the RO and RD of each mRNA. Strikingly, mRNA concentration, which depends on cell physiology and growth conditions, was predicted to be a significant regulatory factor of RO and RD in both growing and nongrowing E. coli cells. This positive effect of mRNA concentration on translation regulation identified at the omics scale was confirmed in a set of selected genes after induction of artificial transcription. Results for E. coli during adaptation to a new carbon source illustrate the physiological relevance of translational regulation by mRNA concentration.

## RESULTS

### Determination of RO and RD for monocistronic genes.

**FIG 1:**
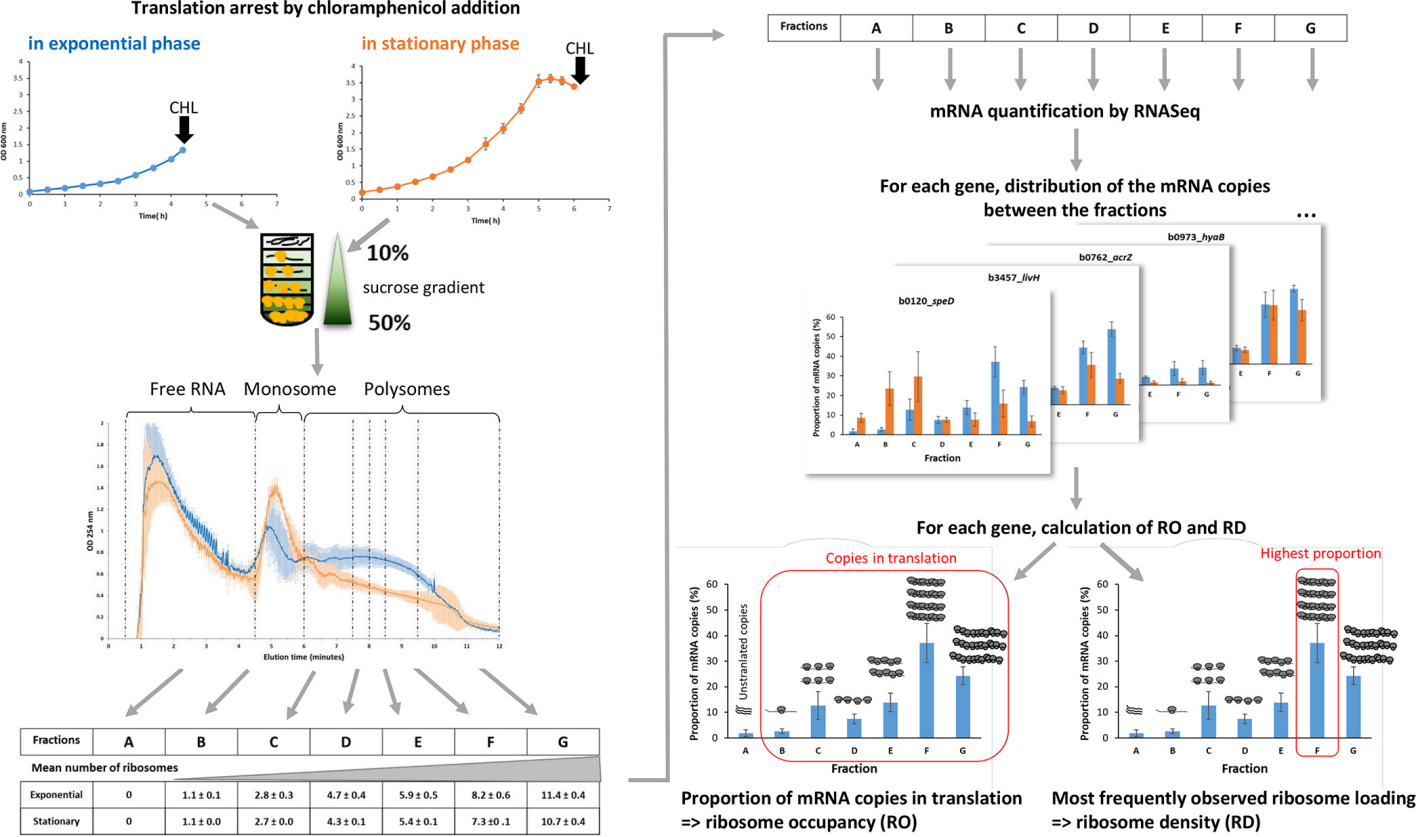
Polysome profiling experiment. All cultures and polysome profiling experiments were repeated three times to provide independent biological replicates in each culture phase. CHL, chloramphenicol.

**FIG 2:**
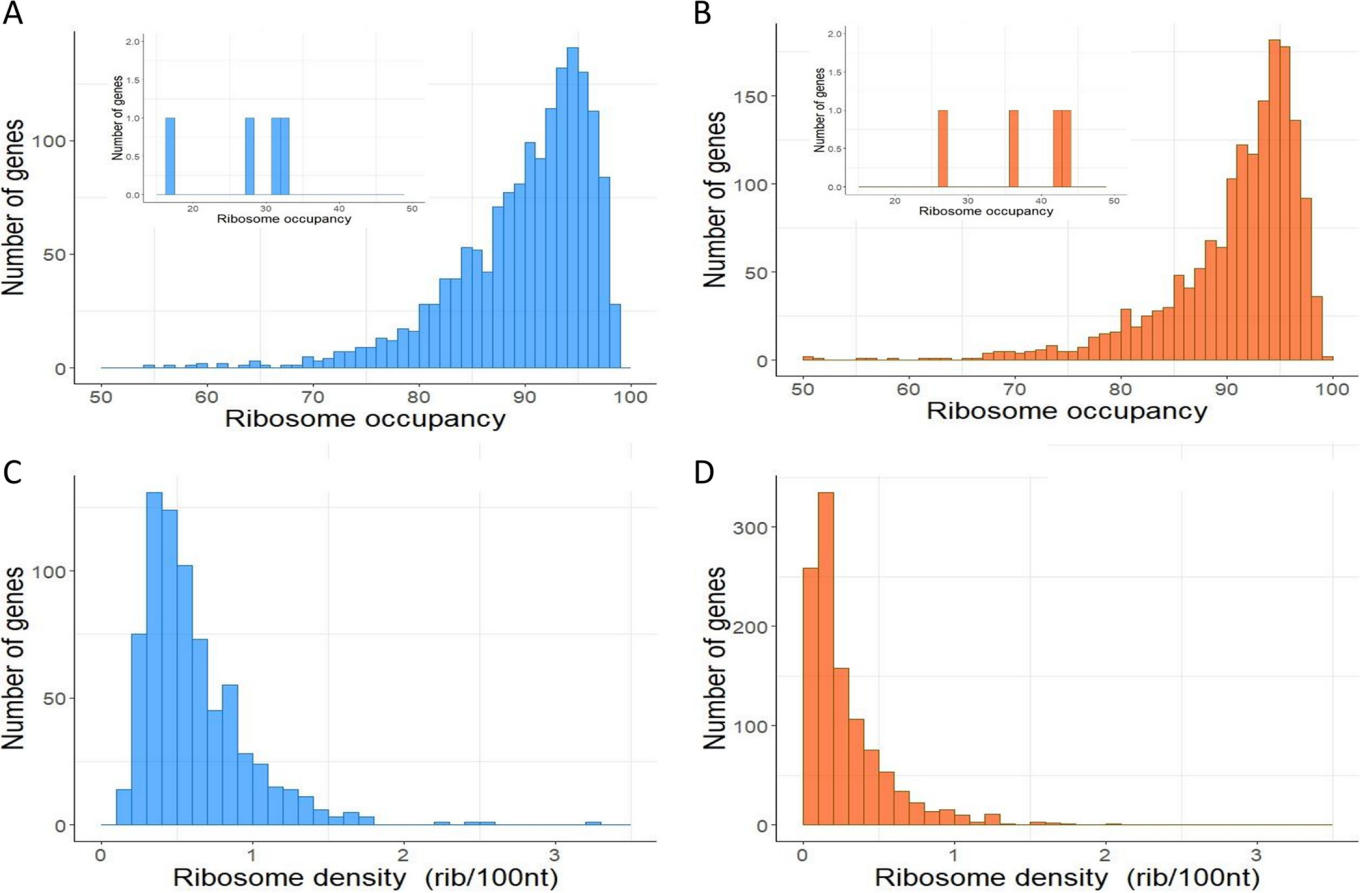
RO and RD for monocistronic genes. Distributions of the mean RO (A) during the exponential-phase for 1563 genes and (B) during the stationary-phase for 1633 genes. The insets are expanded views of the low RO region (between 15% and 50%). RD distribution (C) during the exponential-phase calculated for 732 genes and (D) during the stationary-phase calculated for 1102 genes.

E. coli cells were cultured in the exponential- or stationary-phase (1 h after glucose depletion) to characterize the translational status of E. coli mRNAs in both phases (Fig. 1). The translation was characterized at the genome-wide scale using polysome profiling. mRNA-polysome complexes were divided into seven fractions (A to G) by ribosome load (Fig. 1). The distribution of mRNA copies between fractions for each gene was measured in the two culture phases (Fig. S1). We determined RO, the proportion of mRNA copies undergoing translation, and RD (the most frequently observed number of ribosomes bound on all the different mRNA copies of a gene), for several hundred monocistronic genes in both culture phases (Fig. 2). The analysis excluded polycistronic genes because it is not possible to assign the ribosome load to one or the other cistrons of an operon.

The mean RO values were, respectively, 90% ± 7% and 91% ± 7% in the exponential- and stationary-phases (Fig. 2A and B). These high values indicate that for most genes, nearly all mRNA copies underwent translation. These values are particularly high compared with the 66% value reported for another bacterium, Lactococcus lactis (7).

Genes generally had relatively low RD values with a mean of 0.6 ribosomes/100 nucleotides (nt) in exponentially growing cells (Fig. 2C) and 0.3 ribosomes/100 nt in nongrowing cells (Fig. 2D), which is much lower than the maximum theoretical density of 3.3 ribosomes/100 nt (under the assumption that a ribosome occupies ∼30 nt). The presence of free 30S and 50S ribosomal subunits in fraction A (identified by relative levels of 16S and 23S rRNAs in this fraction [Fig. S2]) indicates that the low RD values were probably not due to a lack of free ribosomes.

We compared the RO and RD values with other translation-related parameters available in the literature for E. coli namely, the RBS score (10), which is widely used to predict translation initiation rates, and the computationally (Transim) predicted translation rate in exponentially growing E. coli cells (22), which account for both translation initiation and elongation regulation. The positive correlations obtained (Table 1) indicate that RO and RD are positively associated with mRNA translation and can be considered translation efficiency metrics.

**TABLE 1:** Simple correlations of RO and RD with RBS score (RBS calculator [10]) and translation rate predicted with Transim (22)^*a*^

Translation-related parameter	Exponential-phase		Stationary-phase	
	RO	RD	RO	RD
RBS score	Coeff = 0.28 pval = 5.9 × 10^−24^ (1210 genes)	Coeff = 0.18 pval = 1.6 × 10^−5^ (583 genes)	Coeff = 0.26 pval = 4.0 × 10^−20^ (1210 genes)	Coeff = 0.20 pval = 1.1 × 10^−8^ (824 genes)
Predicted translation rate	Coeff = 0.26 pval = 7.1 × 10^−20^ (1204 genes)	Coeff = 0.31 pval = 4.3 × 10^−14^ (577 genes)	ND^*b*^	ND

a Spearman correlation coefficients with the associated P values and the numbers of genes in the data set are also listed. Transim modeling was not suitable for the stationary-phase.

b ND, not determined.

### Prediction of regulatory factors for mRNA translation.

**FIG 3:**
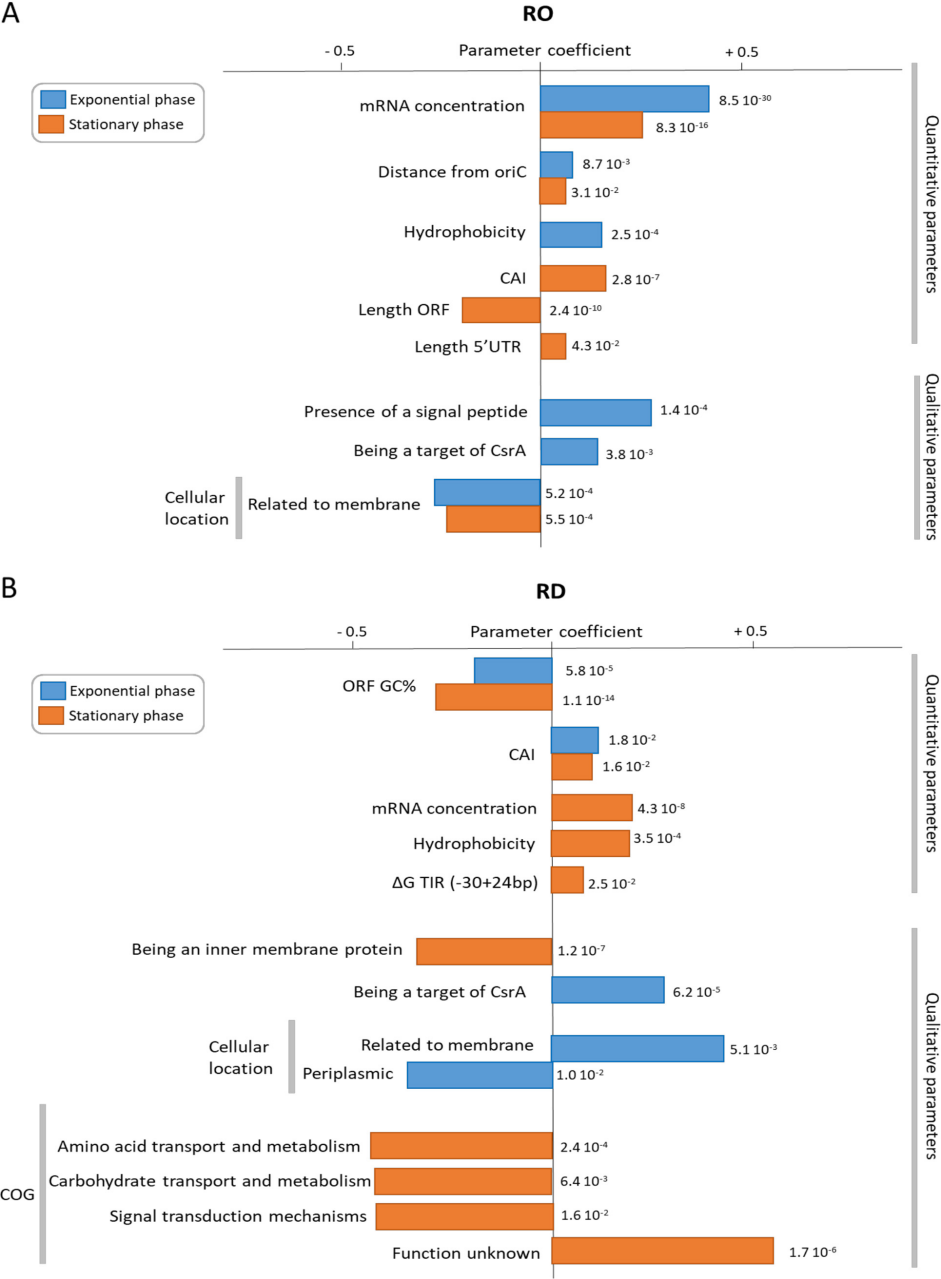
Potential regulatory factors for RO (A) and RD (B) during the exponential- and stationary-phases. Associations in the multiple linear regression models between potential regulatory factors and RO and RD were considered significant at *P* < 0.05. Regression coefficients and *P* values for significant parameters are shown. The influence of quantitative parameters was directly estimated by ranking the absolute values of the associated coefficients. An increase in a parameter with a positive (negative) regression coefficient is associated with an increase (decrease) in the value of RO or RD. For qualitative parameters, the coefficients of one sublevel can only be compared to the coefficients of the other sublevels of the same parameter. In this case, positive (negative) coefficients for a given sublevel mean that the RO or RD value for this level was higher (lower) than the average value of all other sublevels of the same parameter but do not mean that the qualitative parameter had a positive/negative effect on RO or RD.

Factors regulating mRNA translation in E. coli were identified using multiple linear regression. The variability of RO and RD was analyzed and the simultaneous contributions of more than 15 parameters (listed in Materials and Methods) were ranked. Although the coefficients of determination (R^2^) of the models were low (between 0.12 and 0.27), a large set of quantitative and qualitative factors involved in RO and RD regulation during the exponential- and stationary-phases was identified (Fig. 3).

For regulation of the RO, mRNA concentration was a significant positive factor in both culture conditions as well as the distance of the gene from the origin to a lesser extent. In contrast, membrane localization was negatively associated with RO in the exponential- and stationary-phases. Protein hydrophobicity, the codon adaptative index (CAI), the presence of the signal peptide, and being a target of CsrA were positively associated with RO only in one of the two culture conditions. In the exponential-phase, the length of the open reading frame (ORF) was a positive regulator of RO in contrast to the length of the 5’UTR.

Regarding the regulation of RD, the GC% of the open reading frame (ORF) was strongly negatively associated with RD in both culture phases. An increase in the GC% of an ORF, most likely favoring secondary structure formation in the mRNA, was associated with a decrease in RD. This negative effect of sequence folding on RD was also observed in the translation initiation region (TIR) region. The codon adaptative index was positively associated with RD in the two culture conditions. mRNA concentration, protein hydrophobicity, and being a target of CsrA were positive factors of RD in one of the two culture conditions. Metabolic function (the clusters of orthologous groups [COG] parameter) was also associated with RD regulation in a culture phase-specific manner. In the stationary-phase, mRNAs related to amino acid and carbohydrate transport and those with unknown functions had lower and higher RDs, respectively, than those in other functional categories. Results related to protein localization were not so simple: membrane localization was positively associated with RD in the exponential-phase whereas being an inner membrane protein was negatively associated with RD in stationary-phase.

**FIG 4:**
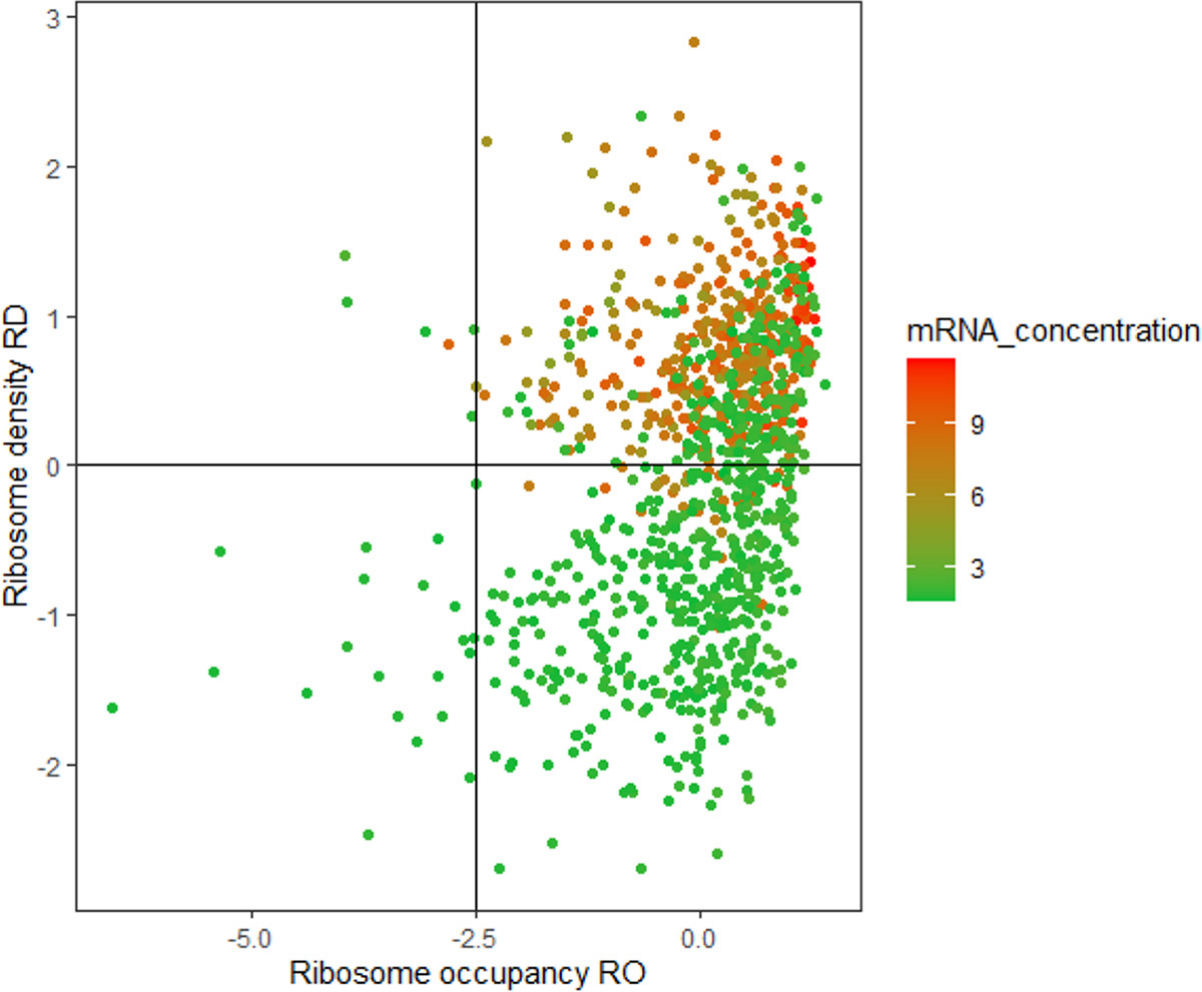
Plot of RD versus RO as a function of mRNA concentration. Data from both culture phases were pooled (1037 values). RD and mRNA concentrations were log-transformed to obtain a normal distribution. All data were centered and reduced.

These results identified the mRNA concentration as a factor regulating translation. Genes with high mRNA concentrations had significantly higher proportions of mRNA copies undergoing translation and higher numbers of the bound ribosome, as illustrated in Fig. 4. mRNA concentration is extremely sensitive to cell physiological state and varies drastically between genes and environmental conditions (>1500 genes are downregulated from the exponential to stationary-phase [23]). Although it is well known that the mRNA concentration directly influences the protein level, the role of mRNA concentration in regulating translation efficiency has not been documented.

### Validation of the effect of mRNA concentration on translation.

**FIG 5:**
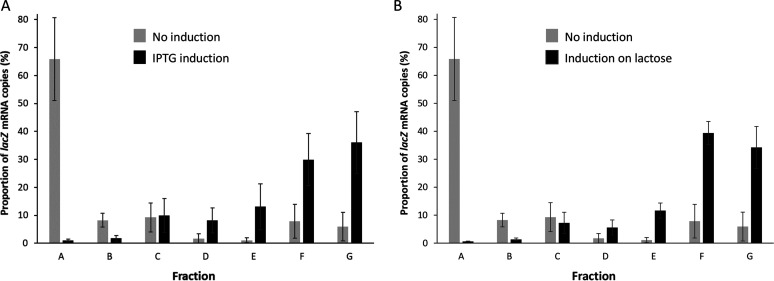
Effect of *lacZ* mRNA concentration on the distribution of mRNA copies between fractions. The distributions are shown for low mRNA concentrations (no induction) and high mRNA concentrations (with induction). Experiments were performed in E. coli MG1655 containing *lacZ* on the chromosome under the native promoter P_Lac._ The noninduced condition corresponds to culture on M9 glucose without IPTG. (A) Artificial *lacZ* induction was performed by adding 1 mM IPTG and (B) physiological *lacZ* induction was performed during growth on M9 lactose. Fraction A consisted of free mRNA molecules not undergoing translation while fractions B to G consist of mRNA copies bound to increasing numbers of ribosomes. Distributions were averaged over two independent biological replicates.

This positive correlation in the genome-wide models between mRNA concentration and RO and RD points toward a relationship between mRNA concentration and translational regulation. To validate this result experimentally, we increased the mRNA concentration of the native chromosomal *lacZ* gene by IPTG (isopropyl β-D-1-thiogalactopyranoside) induction and analyzed the distribution of *lacZ* mRNA copies in the different polysome profile fractions (Fig. 5A). Without induction, most of the mRNA copies were ribosome-free and not undergoing translation (fraction A) leading to a low RO (34% ± 19%). IPTG induction led to a marked decrease in the number of *lacZ* mRNA copies in fraction A, with mRNA copies shifting toward fractions with higher numbers of bound ribosomes (fractions E, F, and G). As a result, the RO of the *lacZ* mRNA increased to 99% ± 1%.

**FIG 6:**
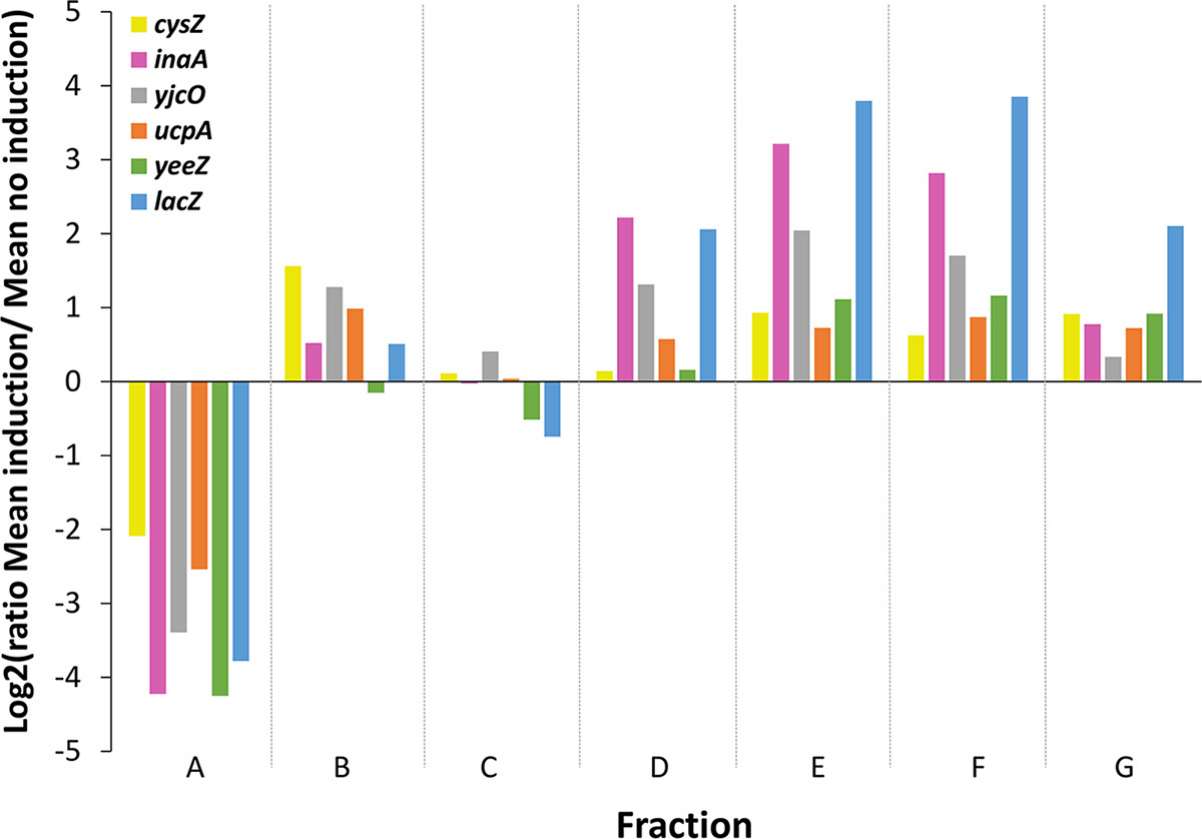
Effect of mRNA concentration on the distribution of copies between fractions for the selected genes. In each fraction, we calculated the log ratio of the mean proportions of mRNA copies at high (after arabinose induction) and low (no induction) mRNA concentrations. Log ratios lower than 0 indicate that the proportion of mRNA copies in that fraction was lower at the high mRNA concentration. Log ratios greater than 0 indicate that the proportion of mRNA copies in that fraction was higher at the high mRNA concentration. Fraction A consisted of free mRNA molecules not undergoing translation, while fractions B to G consist of mRNA copies bound to increasing numbers of ribosomes.

We investigated the generality of this result by analyzing the translational response of six genes (*cysZ*, *inaA*, *ucpA*, *yeeZ*, *yjcO*, and *lacZ*) to changes in mRNA concentration by cloning these genes under the arabinose-inducible promoter. We first used the *lacZ* gene to confirm that the translational responses were similar using a chromosome and a plasmid (Fig. S3). Upon induction with arabinose, the six corresponding mRNA concentrations were increased between 18- and 83-fold (Table S1). Polysome profiling was compared at low and high mRNA concentrations using parallel multiplexed experiments (24), and log ratios of mRNA copy proportions between high and low mRNA concentrations were determined (Fig. 6). For all the genes, the log ratios were lower than 0 in fraction A and higher than 0 in fractions D, E, F, and G, indicating a systematic decrease in the number of free mRNA copies and a shift toward fractions with high ribosome loads at high mRNA concentrations. The RO values increased substantially, by 133%, 61%, 95%, 44%, 37%, and 69% for *lacZ*, *cysZ*, *inaA*, *ucpA*, *yeeZ*, and *yjcO*, respectively. The fact that in the six strains, the concentrations of total RNA, rRNA (16S and 23S), and *rpsJ* and *rplK* mRNAs encoding ribosomal proteins were similar at high and low mRNA concentrations (Table S1) indicated that the observed association between high ribosome load and high mRNA concentration was not due to an increase in the concentration of free ribosomes.

### Physiological role of the translational response to changing mRNA concentration.

To investigate the physiological role of this translational response to changing mRNA concentrations, we studied the adaptation of E. coli to a shift in carbon source from glucose to lactose and compared the translational level of *lacZ* mRNA. These two carbon sources are naturally metabolized by E. coli but lactose metabolism requires the specific induction of *lacZ* to express the β-galactosidase enzyme needed to hydrolyze lactose into glucose and galactose. As expected, both *lacZ* mRNA concentration and β-galactosidase activity were significantly higher on lactose than on glucose (Table S2). Under these conditions, *lacZ* mRNA copies shifted from fraction A (free) toward fractions F and G (heavily loaded with ribosomes; Fig. 5B). As a result, RO increased from 34% on glucose to 99% on lactose. These results are evidence of co-transcriptional regulation of translation in E. coli cells during metabolic adaption to a new carbon source and demonstrate the physiological relevance of this mechanism.

## DISCUSSION

In this work, polysome profiling was used for the first time to obtain a detailed genome-wide picture of translational regulation in E. coli during the exponential- and stationary-phases. Translational regulation was described using RO and RD. The positive correlation of RO and RD with the translation initiation and global translation rates shows that these two variables are good estimators of the translation efficiency of a gene in E. coli. For most genes, nearly all mRNA copies underwent translation regardless of the growth condition (mean RO ∼90%), indicating that translation initiation is efficient in E. coli. However, most mRNAs were loaded with a rather low number of ribosomes. Some studies found that the optimal RD to maximize the protein translation rate was significantly (50% to 80%) lower than the maximal theoretical density (25–26). Higher RDs were predicted to lead to ribosome collisions and traffic that slow down translation (26) and lower RDs to limit translation by the rate of initiation (25). The mean RD of 0.6 and 0.3 ribosomes/100 nt measured in the exponential- and stationary-phases, respectively, represented 18% and 9% of the maximum density. Our findings indicate that translation in E. coli is limited by initiation in both culture conditions for most genes. Although we found that the proportions of ribosomes involved in translation were similar in the exponential- and stationary-phases, the lower RD measured in nongrowing cells may reflect the decrease in the ribosome content associated with the lower growth rate (27). In the stationary-phase, growth-related mRNAs (for amino acid and carbohydrate transport and metabolism) had lower RDs than those in other functional categories. This result suggests that metabolic adaptation to glucose exhaustion involves translational limitation of the synthesis of growth-related proteins. However, the production of proteins with unknown functions is increased, revealing that the physiology of the stationary-phase is still poorly understood.

Exploring the range of RO and RD values within mRNA populations revealed the key factors involved in translation regulation. As expected, these included sequence-related factors. At the omics scale, we observed that the formation of stable secondary structure in the coding sequences (when rich in GC) and TIR region were associated with lower RD. This result is consistent with the negative correlation between *in vivo* ORF secondary structures and the efficiency of protein synthesis per mRNA (28). The positive association between CAI and RO and RD show that abundant codons promote translation efficiency, in agreement with previous reports (12, 13). Regarding the positive correlation between the length of the 5’UTR and RO, we can assume that the longer a 5’UTR is the more likely it is to contain sequences acting as translation initiation enhancers (29). The binding of CsrA was positively associated with RO and RD, but only during exponential growth. CsrA is a well-known activator of the glycolytic pathway in growing E. coli cells (15), which is in agreement with our results.

The cellular localization of the protein was predicted to regulate translation: localization at the membrane was a negative factor of RO in both culture conditions, and positively/negatively associated with RD in the exponential- and stationary-phases. We do not know why membrane protein should be differentially translated and why it should depend on the physiological state. In the literature, controversial results on the link between translation and protein localization were reported confirming the difficulty to conclude on this point (30–32).

**FIG 7:**
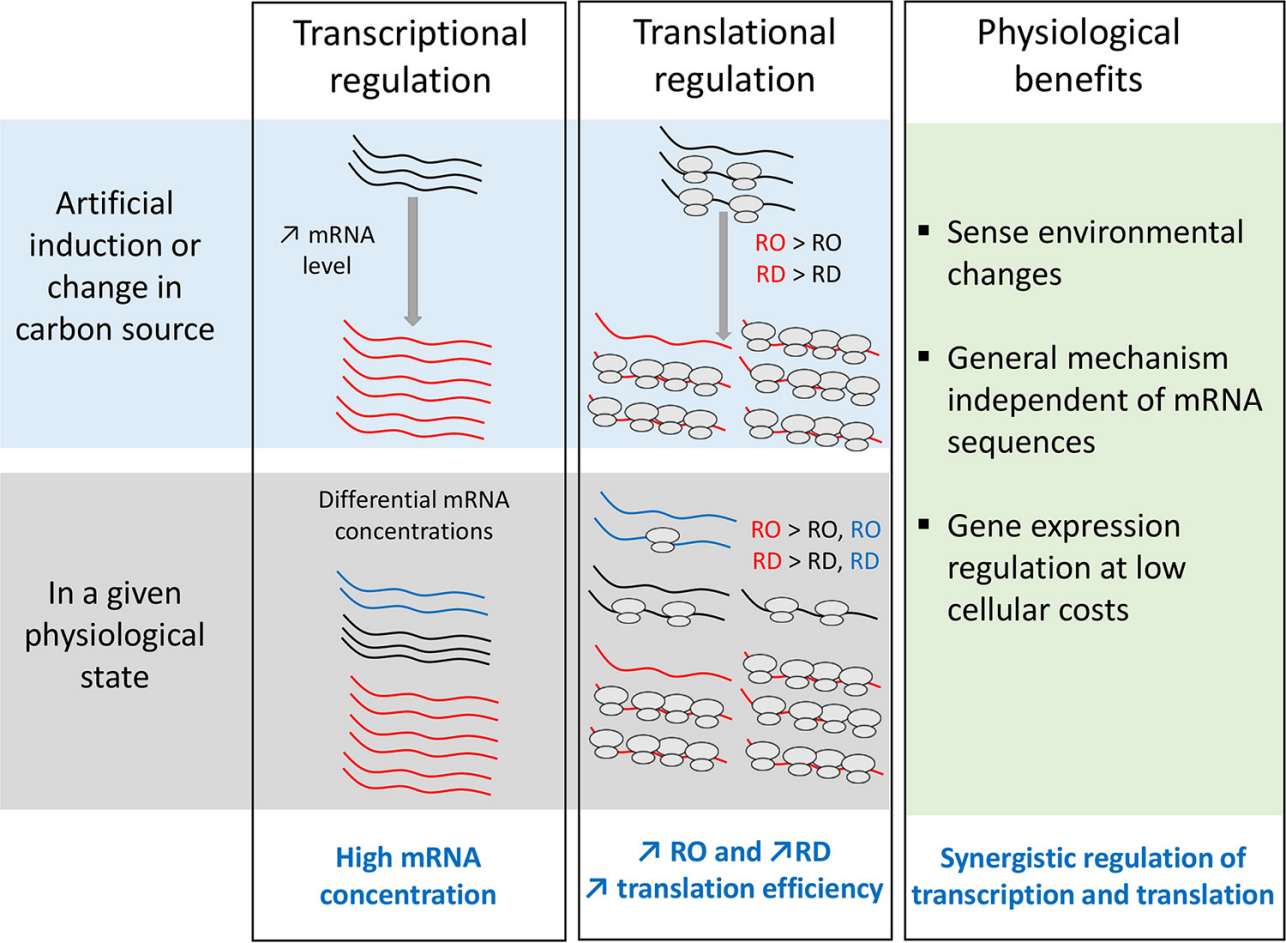
Scheme of the positive correlations between mRNA concentration, RO and RD, and the physiological benefits of the codirectional regulations of transcription and translation.

Unexpectedly, our statistical analyses also identified mRNA concentration as an important positive regulator of both RO and RD at the omics scale in both culture conditions. The relationship between RO, RD, and mRNA concentration has seldom been studied and controversial results were reported in previous studies. mRNA concentration was previously found to have opposed effects on RO and RD in the bacterium L. lactis (7). Using another metric associated with ribosome profiling, the correlation between translation efficiency and mRNA concentration could be either negative in Streptomyces coelicolor and E. coli or positive in another E. coli strain (16, 17). The coupling/decoupling of translation changes from transcription changes could be related to posttranscriptional regulations, spatiotemporal resource limitation for translation, or different rates of transcription and translation (16). In contrast to other studies on the correlation of translation efficiency with mRNA concentration, the positive correlations between transcription and translation regulations were found in two physiological states in our study and experimentally confirmed on selected genes. Furthermore, our results demonstrated the causal relationship in E. coli between an increase in mRNA concentration and higher ribosome occupancy and density (Fig. 7). This is the first reported evidence of codirectional regulation of translation and transcription in E. coli. This coordination is expected to have synergistic effects on gene expression through amplification of the transcriptional signal.

The underlying mechanisms of the positive correlation between mRNA concentration and RO and RD are still unknown. We can speculate based on current knowledge that translating ribosomes may be involved by (i) preventing the backtracking of RNA polymerase and enhancing transcription, (33) and/or (ii) protecting mRNAs against degradation (34) and, thus, increasing mRNA concentrations. Although the contribution of protective ribosomes on a positive correlation between mRNA concentration and RO and RD is yet to be validated, the causality of the effect is reversed because increases in mRNA concentration were found to induce changes in translational regulation and not vice versa. A potential third mechanism could be a competition between mRNAs for ribosome binding. This mechanism considers the interaction between mRNAs and ribosomes as the equivalent of an enzyme-substrate interaction that follows the rules of enzyme kinetics. Such competition has been previously demonstrated between mRNAs for RNase E binding (35, 36), but has yet to be validated in the case of ribosomes.

In new environments, E. coli cells adapt their metabolism by adjusting gene expression. Here, we provide evidence of *in vivo* codirectional transcriptional and translational regulation of *lacZ* when E. coli transitions to growth on lactose. The high β-galactosidase activity required for growth on lactose was achieved through increases in the concentration and ribosome loading of *lacZ* mRNAs. From a physiological point of view (Fig. 7), the coordinated variation in mRNA concentration and translation makes sense as it allows protein expression to be controlled at a low cellular cost. Translational amplification of transcriptional regulation affords high expression levels without high resource expenditure in mRNA synthesis. In addition, this positive correlation between mRNA concentration and the translation may act as a link between translational regulation and cell physiology and growth conditions because mRNA levels are very sensitive to environmental changes. mRNA concentration-mediated translational regulation is a general and easy way for the cell to control gene expression independently of mRNA sequences and without having to express translational regulators such as proteins or sRNAs. This mode of regulation, identified for the first time in this study, is probably important because by coordinating transcriptional and translational regulation, it should have synergistic effects on gene expression and thereby on cell function optimization.

## MATERIALS AND METHODS

### Strains and growth conditions.

For the genome-wide polysome profiling studies, E. coli MG1655 was grown in batch cultures in a bioreactor at 37°C, pH 7.0, and at 350 rpm, in M9 medium supplemented with 3 g/liter glucose as previously described (37). All cultures were inoculated at an optical density at 600 nm (OD_600_) of 0.1 after overnight preculture. The partial pressure of oxygen was continuously monitored during the culture and maintained above 30%. Cells were collected in the exponential-phase at an OD_600_ of ∼1.3 and in the stationary-phase 1 h after glucose depletion at an OD_600_ of ∼3.4.

For polysome profiling study of *lacZ* (coding for β-galactosidase), E. coli MG1655 was grown on M9 medium in a flask at 37°C at 150 rpm. For physiological *lacZ* overexpression, cultures were performed with 8.3 mM lactose as a carbon source, and cells were collected in the exponential-phase at an OD_600_ of ∼1.2. For artificial *lacZ* overexpression, cultures were grown on 3 g/liter glucose with 1 mM IPTG induction when cultures reached an OD_600_ of ∼1 for 30 min.

Strains were constructed to artificially increase the mRNA concentration of specific genes. In addition to *lacZ*, we selected *cysZ* (sulfate transporter), *inaA* (putative lipopolysaccharide kinase), *ucpA* (putative NAD binding oxidoreductase), *yeeZ* (putative epimerase), and *yjcO* (unknown function). For each gene, the 5’UTR + ORF fragment was amplified by PCR and cloned in a P_BAD_/*myc*/His vector (Invitrogen, Carlsbad, CA). Plasmids were introduced into E. coli MG1655 Δ*araFGH*, Ω*pcp18*::*araE533* (38), except for the construct with *lacZ*, which was introduced in E. coli MG1655 *ΔlacZ* Δ*araFGH*, Ω*pcp18*::*araE533* (35). All strains were grown in flasks in M9 medium supplemented with 3 g/liter glucose and 0.1 mg/mL ampicillin at 37°C and 150 rpm. Transcription was induced by adding arabinose (0.001% [wt/vol]) at an OD_600_ of 1 for 30 min. Multiplexing polysome profiling experiments were performed with one experiment on a mix of cell extracts of the six recombinant strains (*cysZ*, *inaA*, *ucpA*, *yeeZ*, *yjcO*, and *lacZ*) obtained without arabinose induction, and one experiment on a mix of cell extracts of the six strains after arabinose induction.

### Polysome profiling experiments.

Classic and multiplexed polysome profiling experiments were performed as previously described (24). Briefly, translation was arrested by adding 0.1 mg/mL chloramphenicol. Cells were harvested, washed twice, and resuspended in lysis buffer (20 mM Tris HCl pH 8, 140 mM KCl, 40 mM MgCl_2_, 0.5 mM DTT, 100 μg/mL chloramphenicol, 1 mg/mL heparin, 20 mM EGTA, 1% Triton X-100). Heparin was used as a RNase inhibitor. After mechanical cell disruption with glass beads, mRNA-ribosome complexes were size-separated on a sucrose gradient (10% to 50% [wt/vol] in polysome gradient buffer [same composition as lysis buffer except for heparin at a final concentration of 0.5 mg/mL]) into 24 subfractions. Total RNA was extracted using the RNeasy Midi kit (Qiagen, Germantown, MD) and quantified using a Nanodrop spectrophotometer (Thermo Fisher Scientific, Waltham, MA). The levels of 16S and 23S rRNAs in each subfraction were calculated using the Bioanalyzer 2100 (Agilent, Santa Clara, CA) and used to pool the subfractions into seven fractions labeled A to G (Fig. S2). Fraction A consisted of subfractions containing DNAs, free RNAs, and free small and large ribosomal subunits. In fractions B to G, the 23S/16S rRNA ratio was constant, at about 1.8, corresponding to entire ribosomes. The 1st peak (fraction B) was attributed to the monosome. The 2^nd^, 3^rd^, and 4^th^ peaks were attributed to 2, 3, and 4 ribosomes, respectively. The number of ribosomes in the other fractions was extrapolated (7). Protein denaturation, nucleic acid precipitation, and total RNA extraction were performed in each fraction as previously described (24).

### RNA quantification by RNA sequencing.

RNA was purified using the RNeasy minikit (Qiagen). rRNA was removed using the Ribo-Zero^RM^ Magnetic kit (Illumina, San Diego, CA). Ribodepleted RNA (10 ng) was then used to construct a sequencing library using the Ion Total RNA-Seq kit v2 kit and the Ion Xpress™ RNA-Seq Barcode 1–16 kit (Thermo Fisher Scientific, Waltham, MA). The library was constructed and (100 bp) single-end sequencing on an Ion Torrent system equipped with an Ion P1 Chip kit was performed by the GeT-BioPuces platform (http://get-biopuces.insa-toulouse.fr/). These experiments were repeated with three independent cultures. Reads were mapped onto the E. coli genome (version U0009.3, GenBank) using the Burrows-Wheeler alignment method (version 0.7.12-r1069) (39). Counting was performed with HTSeq-count version 0.6.1p1 using the intersection nonempty mode. Transcripts with read counts below 10 reads were excluded from the analyses (91 and 21 genes, respectively, in the exponential- and stationary-phases). To correct for experimental variations from ribodepletion to sequencing, the first normalization by library size was performed using the DESeq2 R package. *N_i_*_,_*_j_*_,_*_k DESeq_* represents the count for gene *i* in fraction *j* and replicate *k* after normalizing with the DESeq2 method, with *i* ϵ {1, …, 4497}, *j* ϵ {A, B, C, D, E, F, G} and *k* ϵ {1, 2, 3} in the exponential- and stationary-phases. A second normalization was performed to correct for variations in RNA quantities after ribodepletion because 10 ng of each fraction was used for library construction.

$$N_{i,j,\text{k DESeq}\_\text{ribo}}={{\text{N}}_{i,j,k}}_{\text{DESeq}}\times \frac{\mathrm{ribodepleted}\;\mathrm{RNA}\;\mathrm{quantity}\;j,k}{10}$$

Finally, the variability in the initial amount of total RNA between fractions was considered, and a constant 5 μg of total RNA was used in each fraction for ribodepletion. We obtained the final normalized count for gene *i* in fraction *j* and replicate *k* as follows:

$$N_{i,j,k}={{\text{N}}_{i,j,k}}_{\text{DESeq}\_\text{ribo}}\times \frac{\mathrm{total}\;\mathrm{RNA}\;\mathrm{quantity}\;j,k}{5}$$

For each gene *i*, we calculated the proportion of mRNA copies in each fraction *j* for each replicate *k*:

$$\mathrm{mRNA}\;\mathrm{proportion}\;\mathrm{in}\;{\mathrm{fraction}}_{i,j,k}\left(\%\right)=\frac{N_{i,j,k}}{\sum_{j=A}^{G}N_{i,j,k}}\times 100$$

### RNA quantification by quantitative PCR.

Total RNA (5 μg) was reverse transcribed to yield cDNA using SuperScript II reverse transcriptase (Invitrogen) (40). cDNA was quantified using the Real-Time PCR Detection System (Bio-Rad, Hercules, CA) in 96 well-plates (41). High-throughput quantitative PCR (qPCR) using the Biomark™ HD System (Fluidigm Corporation, South San Francisco, CA) was used when the number of samples to analyze was large (35). To account for the variability between samples and experiments, control Ambion™ ERCC RNA Spike-In Mix was used as an external normalizer (24). A total of eight different genes, two ribosomal RNAs, and four External RNA Controls Consortium (ERCC) spike-ins were quantified (Table S3). *lacZ* mRNA was quantified using the average value obtained from five primer pairs. In unfractionated samples, the concentration of target RNA was estimated as the relative RNA abundance compared to the constant quantity of ERCC spike-ins using the method of fold change ΔCt values (42). In the polysome profiling fractions, the abundance of mRNA was first calculated relative to a constant quantity of ERCC spike-ins and then normalized by the total amount of RNA extracted in each fraction because only 5 μg of the total RNA amount extracted in each fraction was used in the qPCR experiment (24).

### Ribosome occupancy and ribosome density calculations for monocistronic genes.

RO and RD were determined for monocistronic genes using the RegulonDB database. For each gene *i*, RO, the proportion of mRNA copies undergoing translation, was calculated as the total summed proportion of mRNAs bound to at least one ribosome (fractions B to G):

$$\text{R}{\text{O}}_{\text{gene i},k}=\sum_{j=B}^{G}\mathrm{mRNA}\;\mathrm{proportion}\;\mathrm{in}\;\mathrm{fraction}\;(\%{)}_{i,j,k}$$

The final RO for each gene was calculated as the mean RO measured in three replicates in the exponential- and stationary-phases.

The peak fraction (the fraction with the highest proportion of mRNAs undergoing translation) was determined by the bootstrap method on residuals, with a confidence interval fixed at 95% (7). Briefly, the residuals from each average mRNA proportion value, from fractions A to G, were calculated. Then residuals were pooled and reassigned back to these fractions at random to create a bootstrap data set. This residual was added to the mean of the mRNA proportions of each gene to create a bootstrap value of mRNA proportion. One thousand bootstrap data sets were made. The peak fraction was determined in each bootstrap data set analogously to the initial data set. From the one thousand bootstrap data sets, the relative frequency of the highest mRNA proportion within fractions B to G was calculated with a confidence interval fixed at 95%. We only selected genes with a peak fraction confined to a single fraction. The RD per 100 nt was defined as the number of bound ribosomes in the peak fraction normalized to ORF length.

$$\text{R}{\text{D}}_{\text{gene i}}=\frac{\mathrm{number}\;\mathrm{of}\;\mathrm{ribosomes}\;\mathrm{of}\;\mathrm{peak}\;\mathrm{fraction}\;}{\mathrm{Length}\;\mathrm{of}\;\mathrm{Open}\;\mathrm{Reading}\;\mathrm{Fram}e_{i}}\;\text{×}\;100$$

Two genes (*icdC* and *ymgF*) with outlier RDs (>3.3 ribosomes/100 nt) in the exponential-phase were excluded from subsequent analyses. The RD and RO values obtained in the exponential- and stationary-phases are shown in Table S4.

### Multiple linear regression model.

The major regulatory factors for RO and RD were identified using multiple linear regression models with quantitative and qualitative parameters as predictor factors (3, 7, 8). Sets of 873 and 410 genes were included in the models for RO and RD, respectively, during the exponential phase. During the stationary-phase, the sets were 934 and 627 genes, respectively. The models were

$$\mathrm{RO}\;=\text{α }+\beta_{\left[\mathrm{mRNA}\right]}\log\left(\left[\mathrm{mRNA}\right]\right)\;+\;\beta_{\text{ORF length}}\log\left(\mathrm{ORF}\;\mathrm{length}\right)\;+\beta_{\text{ORF GC\%}}\;\mathrm{ORF}\;\mathrm{GC}\%\;+\;{\text{β}}_{\text{CAI}}\log\left(\mathrm{CAI}\right)+\;{\text{β}}_{\text{Chrom}.\text{location }}C\mathrm{hrom}.\mathrm{location}+{\text{β}}_{\text{Hydrophobicity}}H\mathrm{ydrophobicity}\;+\;{\text{β}}_{5\prime U\mathrm{TR}\text{ GC\%}}\;5^{}\prime U\mathrm{TR}\;\mathrm{GC}\%\;+\text{β}{\;}_{5\prime U\mathrm{TR}\text{ length}}\log\left(5^{}\mathrm{\prime U}\mathrm{TR}\;\mathrm{length}\right)\;+\text{β}{\;}_{△GTIR(-30+24\text{bp})}\;\Delta GTIR\left(-30+24\text{bp}\right)+\lambda_{\text{Strand}}\;+{\text{λ}}_{\text{Essentiality}}\;+{\text{λ}}_{\text{Presence signal peptide}}\;+\;{\text{λ}}_{\text{Inner mbr protein}}\;+\;{\text{λ}}_{\text{Cell location}}\;+\;{\text{λ}}_{\text{COG}}\;+\;\lambda_{\text{Target of CsrA}}\;+{\text{λ}}_{2\text{nd }5\text{′UTR}}\;+\text{ξ}$$

$$\log\left(\mathrm{RD}\right)=\alpha +\beta_{\left[\mathrm{mRNA}\right]}\log\left(\left[\mathrm{mRNA}\right]\right)+\beta_{\text{ORF GC\%}}O\mathrm{RF}\;\mathrm{GC}\%\;+\;\beta_{\text{CAI}}\log\left(\mathrm{CAI}\right)\;+\beta_{\text{Chrom}.\text{location}}C\mathrm{hrom}.\mathrm{location}\;+{\text{β}}_{\text{Hydrophobicity}}H\mathrm{ydrophobicity}\;+{\text{β}}_{5^{}\prime \mathrm{UTR}\text{ GC\%}}\;5^{}\prime UTR\;\mathrm{GC}\%\;+\text{ β}{}_{5^{}\prime \mathrm{UTR}\text{ length}}\log\left(5^{}\prime UTR\;\mathrm{length}\right)\;+\text{β}{}_{\Delta \text{G TIR }(-30+24\text{bp})}\;\Delta \text{G }\mathrm{TIR}\;\left(-30+24bp\right)\;+\lambda_{\text{Strand}}\;+{\text{λ}}_{\text{Essentiality}}+{\text{λ}}_{\text{Presence signal peptide}}\;+\;{\text{λ}}_{\text{Inner mbr protein}}\;+\lambda_{\text{Cell location}}\;+{\text{λ}}_{\text{COG}}\;+\lambda_{\text{Target of CsrA}}\;+\lambda_{2\text{nd }5\text{′UTR}}\;+\text{ ξ}$$

where *RO* and *RD* are vectors of the measured levels of the dependent parameter, α is the intercept, β and λ are the coefficients associated with each quantitative and qualitative parameter, and ξ was the error term. Gene-related characteristics (length, GC percentage, strand, chromosome position, distance from OriC, CAI) were obtained or calculated from GenBank (version 3). Gene essentiality and CsrA targets were obtained from Baba et al. (43) and Esquerré et al. (44), respectively. Hydrophobicity was the average GRAVY score (45). Parameters related to protein features (COG annotations and the cellular distributions of proteins) were obtained from the E. coli K-12 genome annotation (46). Inner membrane proteins and proteins with a signal peptide were predicted according to Moffitt et al. (47) and SignalP 4.1 (http://www.cbs.dtu.dk/services/SignalP/), respectively. Genome-wide mRNA concentrations in the exponential- and stationary-phases measured in the same standardized growth conditions have been reported previously (23, 37). Parameters related to 5’UTR and the TIR were also included for genes that exhibited experimentally identified 5’UTRs <300 nt (48, 49). The folding energy of the TIR sequence (−30 + 24 nt) was calculated using the software Mfold. The second 5’UTR is the nature (purine/pyrimidine) of the second nucleotide of the 5’UTR. Positive quantitative parameters were log-transformed when necessary to obtain a normal distribution. Because RD is inversely proportional to ORF length, the ORF length was removed from the RD model. All quantitative parameters were then centered and reduced. The least-squares procedure was used to estimate coefficients of selected parameters and fit quality. Akaike’s information criterion was used to select the model with the best compromise between fitting quality and complexity (3). A *P* value was calculated for each parameter coefficient.

### Simple linear correlation.

Simple linear correlations between two parameters were estimated by calculating Spearman correlation coefficients and corresponding *P* values adjusted for multiple testing using the Benjamini and Hochberg false discovery rate method (50).

### Data availability.

Raw and processed RNA-seq data were deposited in the Gene Expression Omnibus data repository and are accessible through GEO accession GSE191073.
