# Supplementary material for: Synergistic Regulation of Transcription and Translation in Escherichia coli Revealed by Codirectional Increases in mRNA Concentration and Translation Efficiency
Source: Microbiol Spectr. 2022 Feb 9;10(1):e02041-21. doi: 10.1128/spectrum.02041-21 (PMC8826937; doi:10.1128/spectrum.02041-21)
Supplement: SUPPLEMENTAL FILE 2 — Supplemental material. Download SPECTRUM02041-21_Supp_1_seq12.pdf, PDF file, 0.5 MB [file spectrum02041-21_supp_1_seq12.pdf]

Supplemental Material

Figure S1: **Examples of distributions of mRNA copies in fractions A to G during the exponential phase (in blue) and stationary phase (in orange)**. Average values and standard deviations of three independent replicates are shown. Depending on the gene, the distributions between fractions were either similar (first line) or differed (second line) during the exponential and stationary phases.

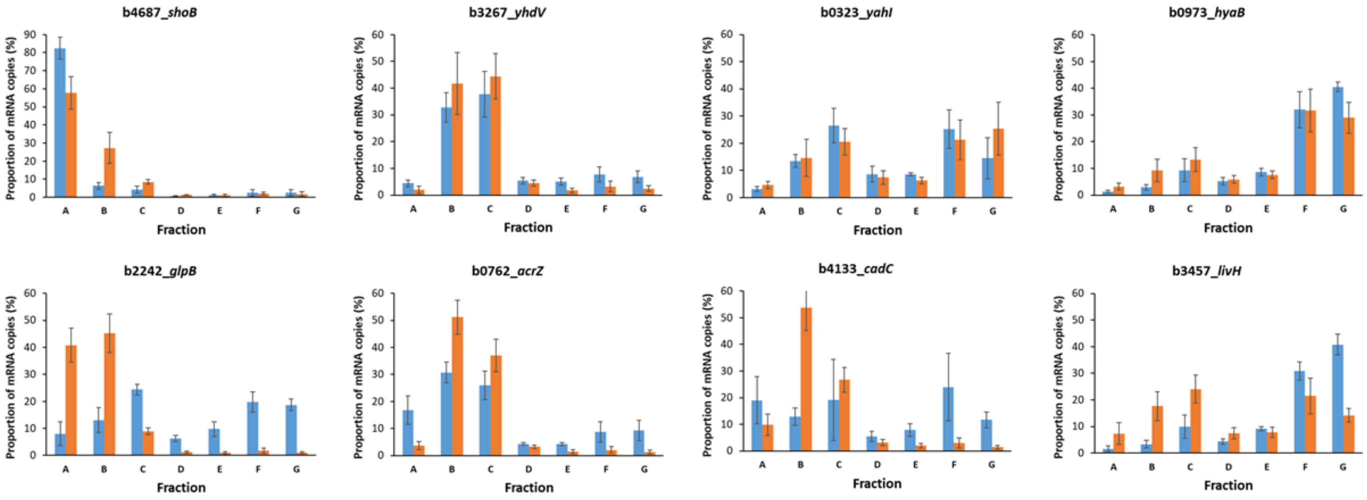

Figure S2: 16S and 23S rRNA levels in subfractions of a polysome profiling experiment performed in exponential phase. 16S and 23S rRNA levels (expressed in percentage of total RNA using the Bioanalyzer) are in blue and orange, respectively. The polysome profile is represented by the gray line. Subfractions are delineated by small vertical dashed black lines. The seven fractions labelled A to G are delineated by long vertical black lines. The position of the 30S ribosomal subunit is identified by the presence of 16S rRNA associated with a low level of 23S rRNA. The position of the 50S ribosomal subunit is identified by the presence of 23S rRNA associated with a low level of 16S rRNA. The 70S and polysome corresponding to entire ribosomes have a constant 23S/16S rRNA ratio at about 1.8.

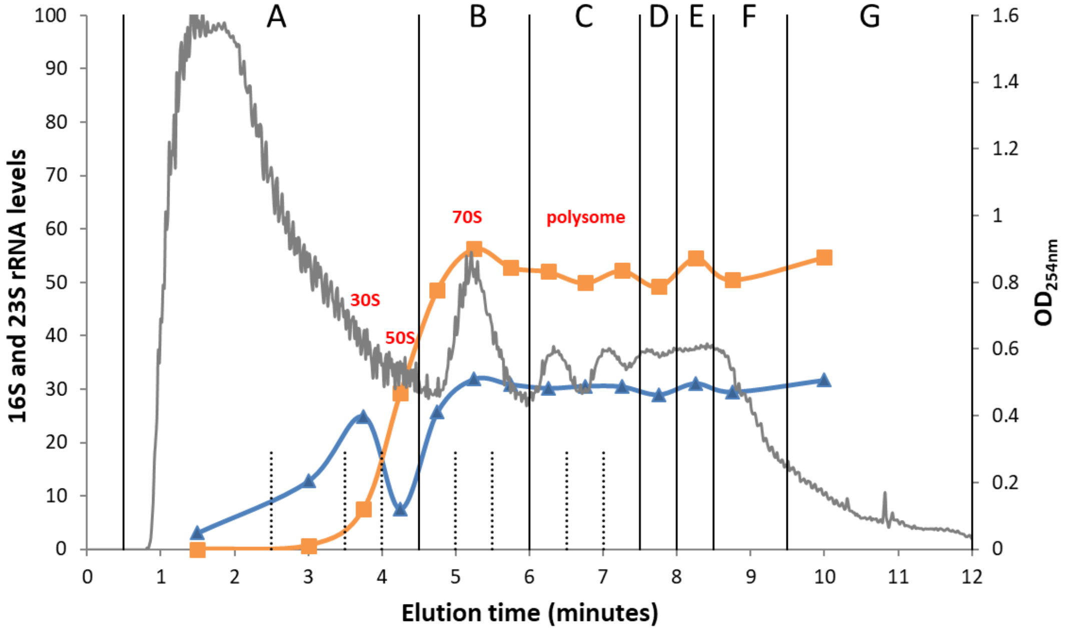

Figure S3: Similar effects of mRNA concentration on the distribution of copies using a chromosome or plasmid *lacZ* gene. Distributions of *lacZ* mRNA copies between fractions were compared between the conditions of low mRNA concentration (no induction) and high mRNA concentration (after induction). (A) Experiments were performed in *E. coli* MG1655 containing *lacZ* on the chromosome under the native promoter $P_{\text{Lac..}}$ The non-induced condition corresponded to a culture without IPTG, whereas induction was achieved with 1 mM IPTG. (B) Experiments were performed in the modified *E. coli* strain containing the *lacZ* gene on a plasmid under the promoter P_{BAD}. The non-induced condition corresponded to a culture without arabinose, whereas induction was achieved with 0.001% arabinose. Fraction A consisted of free mRNA molecules not undergoing translation, while fractions B to G consist of mRNA copies bound to increasing numbers of ribosomes. Distributions were averaged over two independent biological replicates.

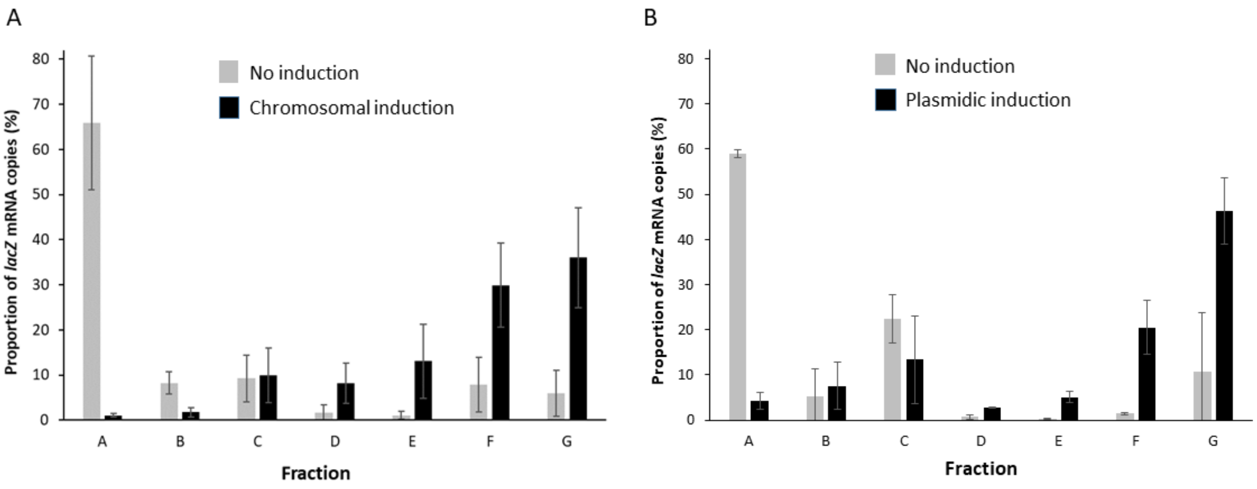

Table S1. Fold-change in concentrations of the induced mRNA, total RNA, ribosomal RNAs (16S and 23S) and ribosomal protein mRNAs (*rpsJ* and *rplK*) in the six recombinant strains after arabinose induction compared to no induction.

	FC induced mRNA	FC total RNA	FC 16S RNA	FC 23S RNA	FC *rpsJ* mRNA	FC *rplK* mRNA
yeeZ recombinant strain	*yeeZ* : 30 ± 7	1.6 ± 0.1	0.7 ± 0.0	1.0 ± 0.0	1.1 ± 0.1	1.2 ± 0.1
inaA recombinant strain	*inaA* : 83 ± 19	0.7 ± 0.1	1.1 ± 0.0	2.2 ± 0.1	1.3 ± 0.0	1.6 ± 0.0
cysZ recombinant strain	*cysZ* : 27 ± 6	1.1 ± 0.0	1.4 ± 0.3	2.0 ± 0.0	1.0 ± 0.1	1.1 ± 0.0
ucpA recombinant strain	*ucpA* : 18 ± 8	0.9 ± 0.1	1.2 ± 0.0	0.3 ± 0.0	1.0 ± 0.1	0.7 ± .0.0
yjcO recombinant strain	*yjcO* : 18 ± 4	0.9 ± 0.0	1.1 ± 0.0	0.6 ± 0.0	1.5 ± 0.0	1.3 ± 0.1
lacZ recombinant strain	*lacZ* : 50 ± 23	1.1 ± 0.0	0.9 ± 0.2	0.9 ± 0.1	0.6 ± 0.1	0.6 ± 0.0
Mean value	/	1.1 ± 0.3	1.2 ± 0.8	1.2 ± 0.8	1.1 ± 0.3	1.1 ± 0.4

Table S2. β-galactosidase activity and *lacZ* mRNA concentration in the different growth conditions. β-galactosidase activity was assayed as previously described (Esquerré et al. (2016). Sci. Rep. 6(1)). Two independent experiments were performed for each assay.

	β-galactosidase activity (mmol/min/g prot)	*lacZ* mRNA concentration
MG1655 on glucose	< 0.05*	0.06 ± 0.02
MG1655 on glucose + 1mM IPTG	6.75 ± 0.03	63.24 ± 1.51
MG1655 on lactose	15.93 ± 0.65	63.21 ± 14.5

*value not determined because it was below the quantification limit of the assay.

Table S3. Sequences of qPCR primers used to quantify mRNAs and ribosomal RNAs and four ERCC RNA spike-ins.

Gene	Function	Forward primer<br>Reverse primer
cysZ	Sulfate transporter	CATCATTCACATCTGCCCCACG<br>GCGCCCCCCATCAACAAAATAT
inaA	Lipopolysaccharide kinase	TGGGCAACAGAGGGCGACTG<br>CGGCCGAACGGATAACGTACG
ucpA	NAD binding oxidoreductase	CTCACGGGCAAGACAGCACTGA<br>TCCGCCAGCTTTTCGATCTCAG
yeeZ	Epimerase	CGGGTTAGGGTGGTTAGGCATG<br>GGCTCCATGCGAAGCAGATAGC
yjcO	Unknown function	TTTGACATTCTTTGCCCACGCC<br>TCAAATCGCCGGAGCTAAACCA
lacZ	β-D-galactosidase	
	pair_1	TCCGTGACGTCTCGTTGCTGC<br>TCACGCAACTCGCCGCACAT
	pair_2	CCCGCATCTGACCACCAGCG<br>CAGCGGCGTCAGCAGTTGTT
	pair_3	GTCGTGACTGGGAAAACCCTGG<br>AACTGTTGGGAAGGGCGATCG
	pair_4	AACAACTTTAACGCCGTGCGCT<br>CACCATGCCGTGGGTTTCAATA
	pair_5	CAGCTGGCGCAGGTAGCAGAG<br>GGCAGATCCCAGCGGTCAAA
rpsJ	30S ribosomal subunit protein	CGTACTCACTTGCGTCTGGTTG<br>AGGCTGATCTGCACGTCTACAC
rplK	50S ribosomal subunit protein	AGCGGCTGGTATCAAGTCTGG<br>GTCATGTCGGCAGCTTTGGTC
16S RNA	Ribosomal RNA	CAAAGGAGACTGCCAGT<br>TCTCTTTGTATGCGCCATTGT
23S RNA	Ribosomal RNA	TCGCTCAACGGATAAAAG<br>GATGAGCCGACATCGAGGTGC
ERCC 130	RNA spike-ins	GTGAAGATGATTGACCGCACGC<br>TGCATATTGCAGCTGAGCCAGC
ERCC 002	RNA spike-ins	CCGTCGGCTGATCGTGGTTT<br>CGACCGTACAGCTCTGGAACCC
ERCC 074	RNA spike-ins	GCCTTGGTAGGGATAGATAGCCACC<br>CTGGGGTTATGAGTAGGGATGAGCA
ERCC 096	RNA spike-ins	CGTAACCAAACATGCACAGCGG<br>TCGCGTCATCGATCCGGGT

Table S4. RO and RD values for monocistronic genes in the exponential and stationary phases. **(Excel file)**

Reference

Esquerré T, Bouvier M, Turlan C, Carpousis AJ, Girbal L, Cocaign-Bousquet M. 2016. The Csr system regulates genome-wide mRNA stability and transcription and thus gene expression in Escherichia coli. Sci Rep 6
